# Characteristics and outcomes of patients with Guillain–Barré syndrome who were admitted to the intensive care unit: a retrospective observational study

**DOI:** 10.1177/03000605241306655

**Published:** 2024-12-24

**Authors:** Samah Alqahtani, Hasan M Al-Dorzi, Hatim Arishi, Ahmad Peeran, Felwa Bin Humaid, Farhan Zayed Alenezi, Jesna Jose, Musharaf Sadat, Naser Alotaibi, Yaseen M Arabi

**Affiliations:** 1 College of Medicine, King Saud bin Abdulaziz University for Health Sciences, King Abdullah International Medical Research Center, Intensive Care Department, King Abdulaziz Medical City, Ministry of National Guard-Health Affairs, Riyadh, Saudi Arabia; 2 College of Medicine, King Saud bin Abdulaziz University for Health Sciences, King Abdullah International Medical Research Center, Neurology Department, King Abdulaziz Medical City, Ministry of National Guard-Health Affairs, Riyadh, Saudi Arabia; 3 King Abdullah International Medical Research Center, King Saud bin Abdulaziz University for Health Sciences, Ministry of National Guard-Health Affairs, Riyadh, Saudi Arabia; 4 Department of Bioinformatics and Biostatistics, King Abdullah International Medical Research Center, King Saud bin Abdulaziz University for Health Sciences, Ministry of National Guard Health Affairs, Riyadh, Saudi Arabia

**Keywords:** Guillain–Barré syndrome, prognosis, intensive care, mechanical ventilation, Guillain–Barré syndrome disability score

## Abstract

**Objective:**

To evaluate characteristics and outcomes in critically ill patients with Guillain–Barré syndrome (GBS).

**Methods:**

Consecutive adults with GBS who required intensive care unit (ICU) admission at a tertiary-care hospital between 1999 and 2020 were enrolled into this retrospective cohort study. Demographics, clinical data and patient outcomes were compared between patients who did or did not receive mechanical ventilation (MV).

**Results:**

During the study period, the number of ICU admissions gradually rose from approximately 900 to 3000 annually. Forty-three patients had GBS and were included, of whom, 27 (62.8%) received MV for a median of 13 days. The MV group stayed longer in the ICU (median, 26 versus 6 days) and in the hospital (median, 120 versus 39 days) than the non-MV group. Most patients in the MV group (22 [81.5%]) required tracheostomy. At maximum follow-up, Hughes Functional Grading scores were 0 (full recovery) in 11 patients (25.5%), 1–3 in 18 (41.8%), 4–5 in 12 (27.9%), and 6 (death) in two (4.6%, both in the MV group), with higher median Hughes score in the MV group (3 versus 0.5). Complications during ICU and hospital stay included: veinous thromboembolism in five (11.6%), gastrointestinal bleeding in three (7.0%), bacteremia in five (11.6%), bedsore in one (2.3%), and GBS-treatment side effects in four (9.4%) patients; all of these complications occurred within the MV group.

**Conclusions:**

GBS was an uncommon reason for ICU admission. The findings highlight significant morbidity with GBS, particularly among patients who need MV.

## Introduction

Guillain–Barré syndrome (GBS) is an acute inflammatory areflexic paralytic polyneuropathy.^1^ The immune-mediated process is classically preceded by an antigenic stimulus, such as viral or bacterial infection.^1,2^ Approximately 30% of patients with GBS require intensive care unit (ICU) admission and mechanical ventilation (MV) secondary to acute respiratory failure caused by progressive motor weakness and diaphragm dysfunction.^3^ Predictors of respiratory failure requiring MV include disability grade on admission, facial or neck weakness, rapid progression of motor weakness, bulbar involvement and autonomic dysfunction,^3–6^ and seasonal variation has been reported.^7–10^ Complications are common in patients receiving MV, occurring in up to 60% of patients, and include pneumonia, sepsis and venous thromboembolism.^11^ These complications may affect recovery.^12–15^ The overall mortality rate of patients with GBS varies between 3 and 7%.^16^ Higher ICU and hospital mortality rates have been reported in older patients, those with significant comorbid illnesses and those who received MV, and may reach up to 20%.^7,12^

Data from Saudi Arabia regarding patients with GBS who were admitted to the ICU are scarce.^17^ In a multicenter retrospective study of 156 patients with GBS admitted to eight tertiary-care centers in Saudi Arabia between 2015 and 2019, 52 patients (33.3%) required ICU admission, of whom 41/156 (26.3%) required MV and 25/156 (16%) required tracheostomy, with a median duration of ICU stay of 17.5 days.^18^ Seasonal variations were reported, with 30.3% of patients admitted during the winter season.^18^

The aims of the present study were to characterize patients with GBS requiring ICU admission, comparing those who required MV with those who did not require MV, and to evaluate hospital outcomes and disability score at the maximum follow-up.

## Patients and methods

### Study population and setting

This retrospective cohort study enrolled all adult patients (aged ≥ 14 years, as defined per local hospital policy) with a diagnosis of new-onset GBS who were admitted to the adult medical-surgical ICUs of King Abdulaziz Medical City, a tertiary-care hospital in Riyadh, Saudi Arabia, during a 22-year period between January 1999 and December 2020. The ICUs operated as closed units and were covered by board-certified intensivists for 24 h per day, 7 days per week. During this period, the bed capacity grew from 21 to 80 beds and the number of admissions from approximately 900 to 3000 annually. GBS was diagnosed by clinical criteria, including acute progressive ascending weakness with areflexia with or without supporting cerebrospinal fluid analysis.^1^ Decisions regarding GBS-related care were made jointly by the ICU and neurology medical teams. The hospital had one inpatient ward for a multidisciplinary rehabilitation program. Patients discharged alive from the hospital were followed-up in the neurology outpatient clinic. All patients with other types of neuromuscular disease causing muscular weakness, or respiratory failure, were excluded from the study.

Ethical approval was obtained from the Institutional Review Board of the Ministry of National Guard Health Affairs – Riyadh (NRC21R/0080/21) and the study was conducted in accordance with the Helsinki Declaration 1975, as revised in 2013. The requirement for informed consent was waived due to the retrospective nature of the study. All patient details were de-identified, and the reporting of this study conforms to the Strengthening the Reporting of Observational Studies in Epidemiology (STROBE) guidelines.^19^

### Data collection

Consecutive patients with new-onset GBS were identified from the prospectively collected ICU database and from electronic and paper-based medical records. The following data were extracted: date of admission, age, sex, history of chronic diseases, history of respiratory or gastrointestinal tract infections before GBS onset, date of symptom onset, relevant clinical presentation including bulbar dysfunction, respiratory distress, or facial weakness, medical research council (MRC) sum score and the modified Erasmus Global Outcome score, reason for ICU admission, and requirement for MV. The MRC sum score is the summation of MRC grades (0 to 5) given for each of 12 muscle groups (left and right upper arm abductors, elbow flexors, wrist extensors, hip flexors, knee extensors, and foot dorsiflexors) and ranges from 0 (paralytic) to 60 (normal strength).^15^ The modified Erasmus Global Outcome score was calculated on day 7 of admission for prognostic prediction of GBS functional outcome at 1 and 6 months. This score evaluates the risk of walking inability based on age at symptom onset (<40 years, score 0; 41–60 years, score 1; >60 years, score 2), preceding diarrhea (absence, score 0; presence, score 1) and MRC sum score on day 7 of admission (60–51, score 0; 50–41, score 3; 40–31, score 6; and 30–0, score 9).^12,13^ The highest possible modified Erasmus Global Outcome score is 12, which estimates the probability of inability to walk at 6 months at 66%.^20^ The electrophysiological testing and documented GBS subtypes (acute inflammatory demyelinating polyneuropathy, acute motor axonal neuropathy variant, and acute motor and sensory axonal neuropathy variant), as determined by the treating neurologist, were reviewed.^21^

In addition, the medical records were reviewed for medical complications that occurred during the ICU stay and in the hospital after ICU discharge, such as: bacteremia, defined as positive blood culture in 2 different sets; hospital-acquired pneumonia; gastrointestinal bleeding (lower/upper); acute kidney injury; venous thromboembolism (deep vein thrombosis and pulmonary embolism); and new bedsore. Hospital-acquired pneumonia was defined as the development of new or progressive infiltrates with associated signs and symptoms of infection (new onset of fever, purulent sputum, leukocytosis, and/or worsening respiratory failure) that required administration of antibiotics or upgrading of ongoing treatment.

Different outcomes were evaluated, including tracheostomy during ICU admission, duration of MV, ICU and hospital mortality, and functional recovery at hospital discharge or at maximal outpatient follow-up after hospital discharge. The GBS disability score (Hughes Functional Grading scale) was also reported at maximal follow-up. This scale assesses the functional status of patients with GBS and is graded as follows:^2,22^ healthy with no signs or symptoms (score 0); minor signs or symptoms of neuropathy but capable of manual work (score 1); able to walk without the support of a stick but incapable of manual work (score 2); able to walk with a stick, appliance, or support (score 3); confined to bed or chairbound (score 4); requiring assisted ventilation (score 5); or dead (score 6).^14^

### Statistical analysis

Patients who received MV (MV group) were compared with those who did not (non-MV group). Statistical analyses were performed using the SAS Statistical Analysis System, version 9.0 (SAS Institute, Cary, NC, USA). Continuous variables are presented as medians and interquartile ranges (Q1, Q3), while categorical variables are presented as frequencies and percentages. Categorical variables were compared between the two study groups using χ^2^-test, or Fisher’s exact test if the number of observations in any of the cells in the 2X2 table were <5. Continuous variables were compared between groups using Student’s *t*-test or Mann–Whitney *U*-test, depending on the normality of distribution. A *P*-value of <0.05 was used to indicate statistical significance.

## Results

### Characteristics of patients

A total of 43 patients with GBS were admitted to the ICU during the study period and were included in the present study. Baseline patient characteristics are outlined in Table 1. The median age was 44 years (interquartile range, 37, 63 years), and 23 patients (53.3%) were male. History of chronic diseases included diabetes mellitus in 10 patients (23%) and chronic cardiovascular disease in 10 patients (23%). A small majority of patients (24 patients, 55.8%) were admitted between the months of October and February (the cold season in Riyadh). A history of preceding infection was documented in 25 patients (58%), with respiratory tract infection reported in 13 (30%) and gastrointestinal tract infection reported in 12 (28%). The median period from symptom onset to hospital admission was 3 days (interquartile range, 1.0, 7.0 days).

**Table 1.** Characteristics and clinical features of patients with Guillain–Barré syndrome who either received or did not receive mechanical ventilation.

Characteristic	Total*n* = 43	MV group*n* = 27	Non-MV group*n* = 16	Statistical significance
Age, years	44.0 (37.0, 63.0)	44.0 (37.0, 64.0)	45.0 (35.0, 62.0)	NS^a^
Male sex	23 (53.5)	13 (48.1)	10 (62.5)	NS^c^
Body mass index, kg/m^2^	26.6 (23.0, 32.5)	26.6 (23.0, 32.2)	26.9 (22.8, 34.1)	NS^b^
Chronic cardiovascular disease	10 (23.3)	8 (29.6)	2 (12.5)	NS^d^
Diabetes mellitus	10 (23.3)	7 (25.9)	3 (18.8)	NS^d^
Days from symptom onset to hospital admission	3.0 (1.0, 7.0)	3.0 (1.0, 5.0)	3.0 (1.5, 14.0)	NS^b^
History of gastrointestinal infection	12 (27.9)	7 (25.9)	5 (31.3)	NS^d^
History of respiratory tract infection	13 (30.2)	8 (29.6)	5 (31.3)	NS^d^
Bulbar dysfunction at admission	12 (27.9)	8 (29.6)	4 (25.0)	NS^d^
Inability to clear airway at admission	6 (14.0)	4 (14.8)	2 (12.5)	NS^d^
Respiratory distress at admission	12 (27.9)	10 (37.0)	2 (12.5)	NS^d^
Facial weakness at admission	11 (25.6)	8 (29.6)	3 (18.8)	NS^d^
Season of presentation				
Cold season, October–February	19 (44.2)	9 (33.3)	10 (62.5)	NS^c^
Warm season, March–September	24 (55.8)	18 (66.7)	6 (37.5)	NS^c^
Physiological parameters at ICU admission				
Glasgow Coma Scale score	15.0 (15.0, 15.0)	15.0 (15.0, 15.0)	15.0 (15.0, 15.0)	NS^b^
Medical research council sum score	25.0 (13.0, 40.0)	18.0 (2.0, 36.0)	36.0 (28.5, 42.0)	*P* = 0.004^b^
Lowest heart rate, beats/min	70.0 (60.0, 82.0)	62.0 (52.0, 88.0)	70.0 (62.5, 80.0)	NS^a^
Lowest systolic blood pressure, mmHg	106.0 (94.0, 119.0)	102.0 (81.0, 112.0)	118.5 (102.0, 128.5)	*P* = 0.005^a^
PaO_2_/FiO_2_ ratio, mmHg	251 (184, 281)	208 (153, 281)	259 (251, 279)	NS^a^
PaCO_2_ level, mmHg	38.0 (34.0, 42.0)	37.0 (34.5, 41.0)	38.0 (34.0, 43.0)	NS^b^
Forced vital capacity, ml/kg	21.3 (13.3, 36.9)	14.0 (7.5, 18.2)	36.9 (24.0, 42.4)	*P* = 0.0002^a^
Maximal negative inspiratory force, cm water	−29.5 (−49.0, –17.0)	−17.0 (–22.5, –7.5)	–49.0 (–60.0, –35.5)	*P* = 0.0009^b^
Modified Erasmus GBS outcome score on day 7 of admission	9.0 (5.0, 10.0)	10.0 (7.0, 11.0)	7.0 (3.0, 9.5)	NS^b^
Laboratory parameters on ICU admission				
Hemoglobin, g/L	93.0 (64.0, 113.0)	88.0 (64.0, 101.0)	115.0 (51.0, 127.5)	*P* = 0.02^b^
Creatinine, µmol/L	60.0 (53.0, 71.0)	61.5 (55.0, 72.0)	60.0 (48.0, 66.5)	NS^b^
Bilirubin, µmol/L	7.0 (5.0, 9.0)	7.0 (5.0, 9.0)	6.9 (5.0, 10.5)	NS^a^
Lactate, mmol/L	1.0 (0.8, 1.1)	1.0 (0.8, 1.1)	1.0 (0.9, 1.5)	NS^b^
International normalized ratio	1.0 (1.0, 1.0)	1.0 (0.9, 1.0)	1.0 (1.0, 1.0)	NS^b^
Treatment during ICU stay				
Vasopressors use	8 (18.6)	7 (25.9)	1 (6.3)	NS^d^
Renal replacement therapy	3 (7.0)	3 (11.1)	0	NS^d^
Tracheostomy	22 (51.2)	22 (81.5)	0	*P* < 0.0001^c^
GBS-specific treatment^e^				
Received intravenous immunoglobulins	38 (88.4)	22 (81.5)	16 (100.0)	NS^d^
Received plasmapheresis	12 (27.9)	12 (44.4)	0	*P* = 0.001^d^

Data presented as median (first quartile, third quartile) or *n* (%) prevalence.

GBS, Guillain–Barré syndrome; ICU, intensive care unit; MV, mechanical ventilation.

^a^Student’s *t*-test; ^b^Mann–Whitney *U*-test; ^c^χ^2^-test; ^d^Fisher’s exact test; ^e^Some patients received more than one GBS-specific treatment.

NS, no statistically significant between-group differences (*P* > 0.05).

Laboratory investigations on admission showed positive polymerase chain reaction result for cytomegalovirus in two patients (4.7%) and Epstein–Barr virus in one patient (2.3%). Testing for *Campylobacter jejuni* was performed in 15 patients (34.9%), *Hemophilus influenzae* in 22 patients (51.2%) and *Mycoplasma pneumoniae* in 34 patients (79.1%); all were negative.

The median MRC sum score at admission was 25 (interquartile range, 13.0, 40.0). Electrophysiological testing was consistent with acute inflammatory demyelinating polyneuropathy in 11 patients (25.6%), acute motor axonal neuropathy variant in 15 patients (34.9%), and acute motor and sensory axonal neuropathy variant present in four patients (9.3%). Eleven patients (25.6%) did not undergo electrophysiological studies, and there were missing records for two patients (4.7%).

### Care in the ICU

#### Respiratory support

A total of 41 patients were admitted to the ICU for a potential need for respiratory support due to signs of impending respiratory failure, including bulbar dysfunction in 12 patients (27.9%), inability to clear airways in six patients (14%), and respiratory distress in 12 patients (27.9%). Facial weakness at admission was reported in 11 patients (25.6%).

Twenty-seven patients (62.8%) received invasive MV. Factors associated with invasive MV included a lower median MRC sum score on admission (18 [interquartile range, 2, 36] versus 36 [interquartile range, 28.5, 42.0] in the non-MV group, *P* = 0.004). In addition, the modified Erasmus GBS Global Outcome scores on day 7 were higher for the MV group than for the non-MV group, with median scores of 10.0 (interquartile range, 7.0, 11.0) versus 7.0 (interquartile range, 3.0, 9.5), respectively (*P* = 0.08).

#### Cardiovascular support

Eight patients (18.6%) required vasopressor support during the ICU stay, mostly among mechanically ventilated patients; seven patients (25.9%) in the MV group versus one patient (6.3%) in the non-MV group. One patient developed multiple episodes of bradycardia with hypotension, with one episode leading to pulseless electrical activity cardiac arrest and one cycle of cardiopulmonary resuscitation before return of spontaneous circulation. He was evaluated by a cardiac electrophysiologist and recovered without pacing.

#### GBS-specific treatments

Among the study cohort, 38 patients (88.4%) received intravenous immunoglobulins, with comparable between-group prevalence (22 [81.5%] in the MV group and 16 [100%] in the non-MV group), and 12 patients (27.9%) received plasmapheresis (all in the MV group).

### Complications

A total of 18 patients (42%) developed complications during their hospital stay, mostly while in the ICU (summarized in Table 2). The complications during ICU stay included hospital-acquired pneumonia in 14/27 patients (51.9%) in the MV group versus one/16 patients (6.3%) in the non-MV group (*P* = 0.002). Venous thromboembolism was diagnosed in five patients (11.6%), all within the MV group, deep vein thrombosis in three patients (7.0%), and pulmonary embolism in three patients (7.0%, one of whom had both deep vein thrombosis and pulmonary embolism). Gastrointestinal bleeding occurred in three patients (7.0%); one patient required blood product transfusion (hemoglobin level dropped to 69 g/L). No invasive intervention was needed. Other complications included bacteremia in five patients (11.6%), candidemia in two patients (4.7%), and bedsores in one patient (2.3%).

**Table 2.** Complications and outcomes of patients with Guillain–Barré syndrome who either received or did not receive mechanical ventilation.

Characteristic	Total*n* = 43	MV group*n* = 27	Non-MV group*n* = 16	Statistical significance
Complication during ICU stay				
Hospital-acquired pneumonia	15 (34.9)	14 (51.9)	1 (6.3)	*P* = 0.002^b^
Line-related complication	3	2 (7.4)	0	NS^c^
Treatment-related complication	4 (9.3)	4 (14.8)	0	NS^c^
Acute kidney injury	7 (16.3)	7 (25.9)	0	*P* = 0.03^c^
Any venous thromboembolism	5 (11.6)	5 (18.5)	0	NS^c^
Deep vein thrombosis	3 (7.0)	3 (11.1)	0	NS^c^
Pulmonary embolism	3 (7.0)	3 (11.1)	0	NS^c^
GI bleeding (lower/upper)	3 (7.0)	3 (11.1)	0	NS^c^
Bacteremia	5 (11.6)	5 (18.5)	0	NS^c^
Bedsore	1 (2.3)	1 (3.7)	0	NS^c^
Complication post ICU discharge until hospital discharge				
Deep vein thrombosis	2 (4.7)	2 (7.4)	0	NS^c^
Pulmonary embolism	2 (4.7)	2 (7.4)	0	NS^c^
GI bleeding (lower/upper)	2 (4.7)	2 (7.4)	0	NS^c^
Bedsore	1 (2.3)	1 (3.7)	0	NS^c^
Clinical outcome				
Hospital length of stay, days	86.0 (33.0, 144.0)	120.0 (70.0, 174.0)	39.0 (11.0, 91.5)	*P* = 0.0007^a^
ICU length of stay, days	22.0 (6.0, 35.0)	26.0 (22.0, 42.0)	6.0 (3.0, 6.0)	*P* < 0.0001^a^
Discharge from hospital alive	41 (95.3)	25 (92.6)	16 (100.0)	NS^c^
Mechanical ventilation duration, days	13.0 (11.0, 17.0)	13.0 (11.0, 17.0)	0	
Functional outcome at follow-up, Hughes score^d^	3.0 (0.0, 4.0)	3.0 (3.0, 5.0)	0.5 (0.0, 3.0)	*P* = 0.0005^a^
Favorable functional outcome (score 0–2)	16 (37.2)	5 (18.5)	11 (68.8)	*P* = 0.003^a^
Unfavorable functional outcome (score 3–5)	25 (58.1)	20 (74.1)	5 (31.2)	*P* = 0.01^a^
Death (score 6)	2 (4.7)	2 (7.4)	0 (0)	NS^c^

Data presented as median (first quartile, third quartile) or *n* (%) prevalence.

GBS, Guillain–Barré syndrome; GI, gastrointestinal; ICU, intensive care unit; MV, mechanical ventilation.

a Mann–Whitney *U*-test; ^b^χ^2^-test; ^c^Fisher’s exact test.

d Hughes score (Hughes Functional Grading scale [GBS disability score]): score 0, healthy with no signs or symptoms; score 1, minor signs or symptoms of neuropathy but capable of manual work; score 2, able to walk without the support of a stick but incapable of manual work; score 3, to walk with a stick, appliance, or support; score 4, to bed or chairbound; score 5, requiring assisted ventilation; and score 6, dead.

NS, no statistically significant between-group differences (*P* > 0.05).

Treatment-related complications after intravenous immunoglobulin infusion were observed in four patients (9.4%) and included nausea/vomiting in three patients (7.0%) and hyperthermia (highest temperature recorded being 38.4 °C) in one patient in the first 24 h of intravenous immunoglobulin infusion. None of these complications were observed in the non-MV group.

### Outcomes

The median duration of MV was 13 days (interquartile range, 11, 17 days). Tracheostomy was performed for 22 out of the 27 patients who received MV on a median of 15 days (interquartile range, 11.0, 19.0 days) after ICU admission. The median ICU stay was 22 days (interquartile range, 6, 35 days), and was significantly longer in the MV group than the non-MV group (26 days [interquartile range, 22.0, 42.0 days] versus 6.0 days [interquartile range, 3.0, 6.0 days]; *P* = 0.0001). The median hospital stay was also significantly longer in the MV group than the non-MV group (120 days [interquartile range, 70, 174 days] for the MV group versus 39 days [interquartile range, 11, 91.5 days] for the non-MV group; *P* = 0.0007). Death occurred in two patients (4.7%), both of whom received MV and died during ICU stay (Table 2).

The GBS disability score (Hughes scale) was calculated for all patients with a median follow-up of 13.2 months (interquartile range, 0.1, 57.0 months). For nine patients, the maximum follow-up was possible only to hospital discharge, as they were transferred to other institutions. The median Hughes' scale score was 3 (interquartile range, 0, 4), with 16 patients (37.2%) having a score of 0–2 (favorable functional recovery; 11 had full recovery to baseline), and 25 (58.1%) having a score of 3–5 (unfavorable functional outcome). The MV group had higher scores (median of 3 [interquartile range, 3, 5] versus a median of 0.5 [interquartile range, 0.0, 3.0] in the non-MV group; *P* = 0.0005) indicating less favorable functional outcomes (Table 2).

## Discussion

In the present study, GBS was an uncommon reason for ICU admission, with the main indication being the need for respiratory support. GBS was associated with substantial morbidity, particularly among mechanically ventilated patients, and intravenous immunoglobulins were commonly used for treatment. The overall hospital mortality rate in patients with GBS admitted to the ICU over the 22-year study period was 4.7%. Only one in four patients had no disability at follow-up.

Impending respiratory failure is a common cause of admission to the ICU in patients with GBS, as observed in the current and previous studies.^3,16^ Early recognition of impending respiratory failure and admission to the ICU is important to prevent respiratory distress, aspiration, and cardiopulmonary arrest. In the present study, lower muscle strength, forced vital capacity, negative inspiratory force, and modified Erasmus Global Outcome score were found to be associated with the requirement of MV support. Forced vital capacity less than 60% is a predictor of respiratory failure and is frequently used to monitor patients with GBS.^4^ Additionally, lower vital capacity (13.0 ± 5.9 ml/kg versus 21.9 ± 8.4 mL/kg, *P* = 0.003) has been shown to correlate with reintubation and possibly tracheostomy.^23^ In the present study, the inability to clear secretions and bulbar involvement were similar between patients who received or did not receive MV. Without intubation, such patients require significant nursing and respiratory care, which may not be available in all ICUs.

In the current study, intravenous immunoglobulins were more commonly used than plasmapheresis for the treatment of GBS. Both treatment modalities were available at King Abdulaziz Medical City during the study period and have been shown in clinical studies to be equally effective, although plasmapheresis may be associated with lower direct costs that intravenous immunoglobulins.^1,24,25^ In the present hospital, there is a general preference to use intravenous immunoglobulins, and plasmapheresis is usually avoided in patients with autonomic dysfunction.^1^

The present study demonstrated that complications were common during the course of GBS in the ICU, the most important being hospital-acquired pneumonia, central line-associated bloodstream infection and venous thromboembolism. Such complications are largely preventable. Delaying intubation may increase the risk of early-onset pneumonia,^2^ however, a randomized controlled trial of early MV via face mask or endotracheal intubation, due to the presence or absence of impaired swallowing (experimental arm) versus conventional care, found no difference in pneumonia (10/24 [40%] versus 9/25 [36%], respectively; *P* = 1.0).^26^ Nevertheless, interventions such as the ABCDEF bundle, which incorporates evidence-based guidelines for the prevention and management of pain, agitation/sedation, delirium, immobility, and sleep disruption, are warranted and have been associated with improved outcomes in general ICU patients.^27^

In the current study population, there were two in-hospital deaths (4.7%), and both patients had received MV. GBS-associated mortality rates have been reported to vary between 3 and 7%,^16^ with higher mortality rates observed in patients who received MV.^7,12^ A population-based study from Denmark showed that the short-term (0–6 months) mortality was 4.8% (95% confidence interval: 4.0, 5.8), and the intermediate-term (7–48 months) mortality was 7.6% (95% confidence interval: 6.5, 8.9).^28^ In another study from Taiwan, the predictors of hospital mortality included older age, endotracheal intubation, MV, cardiac complications, and systemic infection.^29^ In the present study, only a minority of patients had complete recovery at follow-up. This is consistent with other studies, demonstrating that significant disability after acute illness is not uncommon. One study from Korea found that 10.2% of patients had moderate to severe physical disability at a median follow-up of 6 months,^30^ with older age, a long hospitalization period, and tracheostomy found to be associated with increased disability.^30^ Axonal variants of GBS, which were common in the present study population, may also be related to poor recovery.^31^

The present study results should be interpreted taking into consideration potential limiting factors. Data were collected retrospectively from electronic and paper-based medical records predisposed to missing data. The maximum follow-up for some patients was only at hospital discharge, as they were discharged early to specialized rehabilitation and physical therapy hospitals for further management, and follow-up with them was lost. Finally, although the study period extended over 22 years, the sample size was small, reflecting the uncommon nature of GBS.

## Conclusions

In the present study, patients with GBS who were admitted to the ICU and required MV had a higher risk of developing complications during the ICU and hospital stay, prolonged length of stay, and worse functional recovery. The poorer outcomes and more frequent complications associated with MV in the present study are likely related to GBS disease severity. Prospective studies are needed to further evaluate the outcomes of critically ill patients with GBS.

## Data Availability

The datasets generated during and/or analyzed during the current study are not publicly available but will be made available from the corresponding author upon reasonable request.
